# Knee osteochondritis dissecans-treatment technical aspects

**DOI:** 10.1016/j.jor.2022.08.005

**Published:** 2022-08-11

**Authors:** Mats Brittberg

**Affiliations:** Cartilage Research Unit, University of Gothenburg, Region Halland Orthopaedics, Varberg Hospital, S-43237, Varberg, Sweden

**Keywords:** Osteochondritis dissecans, Cartilage repair, Osteochondral graft fixation, Osteochondral repair

## Abstract

**Purpose and objective:**

Current treatments of different stages of knee osteochondritis Dissecans (OCD) are depending on the age of the patients and the stability of the diseased osteochondral area. The purpose of this paper was to summarize the treatment alternatives in order to simplify the choice for the treating surgeon.

**Background and principle results:**

Osteochondritis dissecans (OCD) of the knee is an idiopathic and local osteochondral abnormality that affects mainly children and adolescents with risk of loosening of osteochondral fragments. A good clinical result can be expected when the physes are still open, when the osteochondritis is small and when the osteochondritis can be assessed as stable by MRI. Unstable OCD lesions most often need to be treated operatively by different fixation methods and when the osteochondral cannot be refixated, different local chondral and osteochondral repairs are available to fill up the defect area to congruity

**Summary and major conclusions:**

The final choice of which treatment to use is depending on fragment viability and forms. Viable fragments are refixated while poor quality fragments are removed followed by a local biological osteochondral repair. Such osteochondral resurfacing may be single bone marrow stimulation with or without scaffold augmentation or different cell seeded grafts.

## 1 Introduction

Osteochondritis dissecans (OCD) is characterized by an aseptic necrosis of a localized area of the subchondral bone. The necrosis then occurs in the epiphysis in its subchondral bone so that a demarcation zone of isolated bone with overlying cartilage is produced. Finally, that diseased area may become loose and give rise to mechanical symptoms.^1^ OCD injuries are different from those that are classified as osteochondral fractures but the radiographs and symptom picture may be similar.

This paper is focused on the surgical management of OCD.

## 2 Diagnostics

### 2.1 Symptoms

Initial symptoms are often diffuse and when the cartilaginous bone fragment start to become separated from the surrounding cartilage then the patient feels pain, tenderness, locking and swelling phenomena. Sometimes the patient could get a feeling of instability and snapping and crepitation are common.^2^^,^^3^

### 2.2 Imaging diagnostics

#### 2.2.1 Plain x-ray

The type of radiology first of all to include are common long weight bearing standing knee images with anterior-posterior images as well lateral images with the knee flexed 35° and a 45° with a ¨patella sunrisë image.

A lateral view can demonstrate an OCD as well as compression fractures where the cortex is irregular. If one suspects an OCD, it is valuable to also take an intercondylar ¨notch¨ view, popularly called ¨tunnel view¨.^4^^,^^5^

A presence of a sclerotic zone around the change can be used to predict the OCD change. Absence of sclerosis ring speaks may indicate that such a lesion could spontaneously heal with conservative treatment.^6^

However, contrary, occurrence of a sclerotic edge indicates risk of progression, that the lesion is not healed and that an operative treatment may be indicated.

-Magnetic Resonance Imaging-MRI.

The next step is to include is a MR examination as it could even better tell us about the stability of the OCD region.^7^

#### 2.2.2 Signs of instability are

• A high signaling line in the boundary between the OCD fragment and surrounding bone.
• Synovial fluid visible between fragment and surrounding bone.
• 5 mm or larger fluid-filled cysts under the fragment.

However, there are certain difficulties as a high signal behind the OCD fragment could also indicate presence of granulation tissue which implies a healing reaction. The symptoms will then be our evaluation instrument and a patient with pain and signal changes surrounding the fragment therefore may need an arthroscopy examination for an instrumental direct testing of fragment stability.^8^

In order to support the treatment choice and when to follow up treatment results, use of classifications may be useful. One such MRI classification has been described by Diapola et al.^9^:

Grade I: No cracks or breakthroughs through the cartilage layer. Cartilage layer is slightly thickened.
Grade II: Cracks with breakthrough of the cartilage layer. Low signaling zone behind the fragment is indicating fibrous anchorage.
Grade III: Cartilage breakthrough with high T2 signal changes behind the fragment indicating fluid behind the fragment and a potentially unstable fragment.
Grade IV: Osteochondral loose fragment with underlying cartilage-bone defect.

#### 2.2.3 Computed tomography and SPECT

Computed tomography with three-dimensional reconstruction of bone involvement is of great importance^10^ when to use as a supplement to MRI especially when to assess degree of bone filling after bone grafting.^11^ A 3D reconstruction is also good to use when to get a more exact size and position of the lesion. See Fig. 1A, Fig. 1BA–B.

**Fig. 1A:**
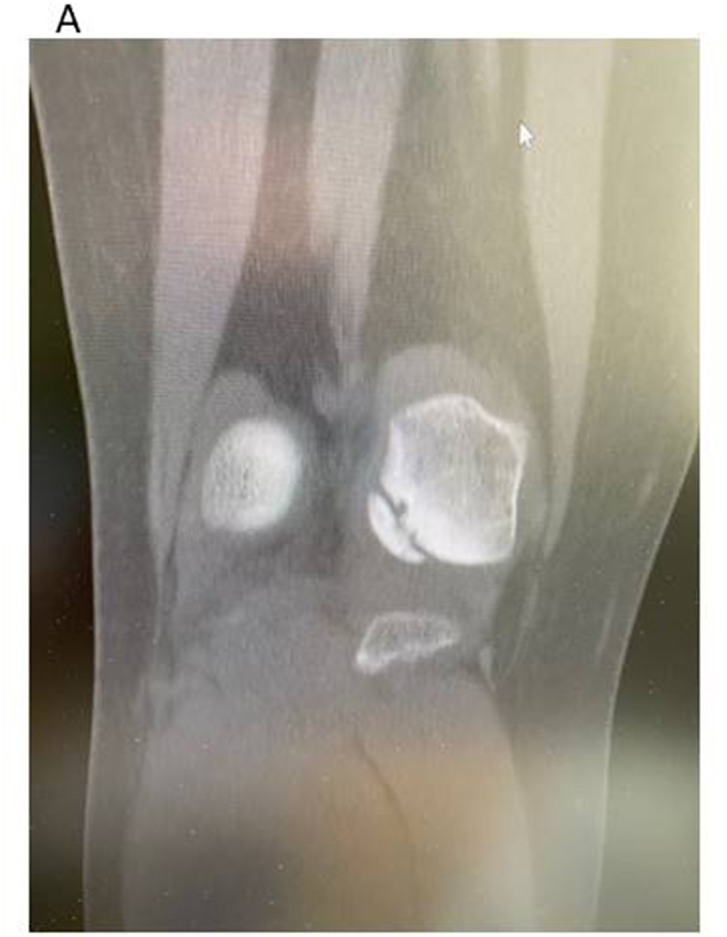
A medial femoral condyle lesion seen on CT scan image. It is obvious that it is loose with no bony contact.

**Fig. 1B:**
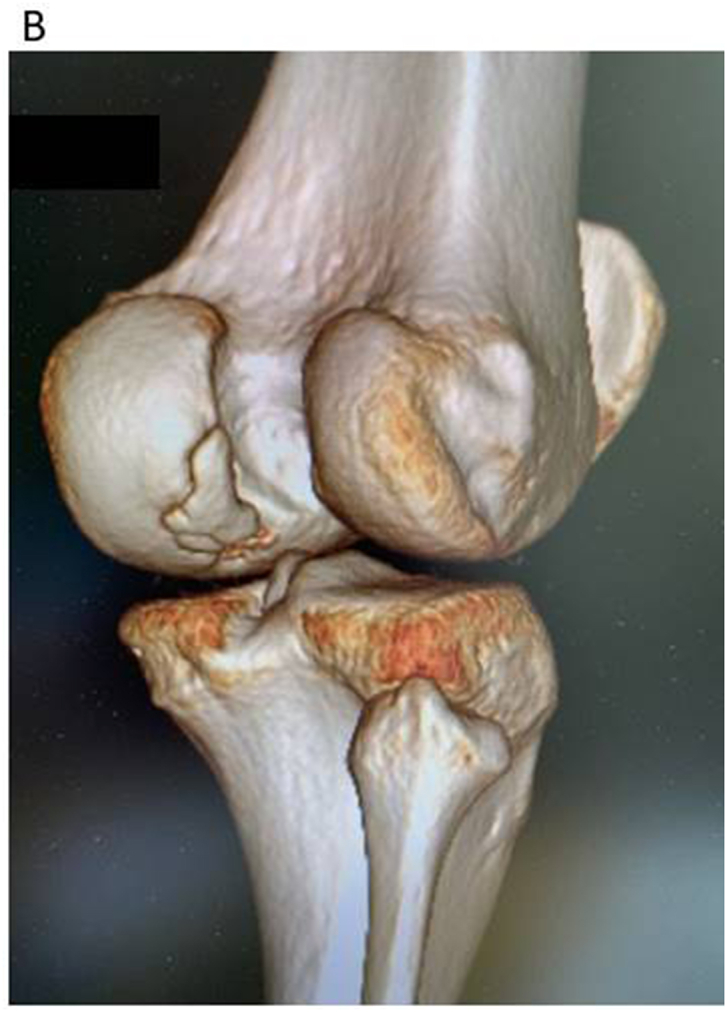
The same lesion as on Fig. 1A but now seen from a view possible to get with a 3D CT scan reconstruction.The exact size could be calculated from such a reconstruction and also the reachability.

Another interesting method is SPECT^12^ which stands for Single photon emission computed tomography and involves capturing gamma radiation from the patient's body through a 360-degree gamma camera. SPECT can be used to further assess bone healing.^12^

Furthermore, Positron emission tomography (PET)^13^ utilizes molecules labeled with a positron-emitting radionuclide. With this method one can study bones the metabolism.^13^

#### 2.2.4 Scintigraphy-isotope examination with radioactive technetium^14^

Skeletal scintigraphy is better than usual X-ray examinations when to diagnose an avascular necrosis. A classification on how to assess scintigraphic uptake in juveniles OCD patients has been described by Cahill and Berg^15^, see below.

**Cahill and Berg's classification of scintigraphic uptake in juvenile osteochondritis Dissecans**.

Grade 0: Normal x-ray and normal scintigraphic examination
Grade 1. The injury is visible on plain radiographs but with normal scintigraphy.
Grade 2. The scintigraphy shows increased absorption at the injury.
Grade 3. In addition to local uptake at the injury, there is also a generally increased uptake throughout the condyle.
Grade 4. As in 3, but here also increased absorption on the opposite side in the joint and in the tibia

### 2.3 Arthroscopic examination

To facilitate the assessment and above all to be able to describe more easily the appearance of the injury, different arthroscopic classifications have been presented. The most well-known classification has been described by Guhl 1982.^16^

Stage 1: Stable osteochondritis
Stage 2: Osteochondritis showing signs of early separation from surrounding bone.
Stage 3: Partially loose osteochondritis partially detached from surrounding bone.
Stage 4: Crater with loose osteochondral complete fragment or several segments of osteochondritis.

A more surgical directed classification has been presented by ICRS (International Cartilage regeneration and preservation society).^17^ It is a modified Guhl classification.

ICRS OCD Grade 0 = Stable osteochondritis with normal overlying cartilage.

• **ICRS OCD Grade I:** Stable osteochondritis with complete cartilaginous layer but with
  areas of softened cartilage upon indentation.
• **ICRS OCD Grade II:** Stable osteochondritis but with partial discontinuity and cracking partial to surrounding cartilage.
• **ICRS OCD Grade III:** The osteochondritis is still in place but with total discontinuity to surrounding cartilage layer.
• **ICRS OCD Grade IV:** Empty cartilage defect where the osteochondritis has completely detached and lies in a free joint like a free body.

Before the treating doctor decides the treatment of an OCD, the natural course of an OCD must be understood. The natural history is directly dependent on the patient's age at start of symptoms. In a patient with juvenile Osteochondritis Dissecans (patient with completely open distal femoral physis) the prognosis is excellent if the cartilage damage is an ICRS Grade I-II. Most juvenile OCD heals without any residual problems if they belong to that the category without dislocation of fragments and classification ICRS Grade I-II.^18^

In a patient in the transition between juvenile and adult stages, a state that we call adolescent stage it is more difficult to give a secure prognosis.

However, the adult type where the patient's physis has been closed has a worse prognosis. Here it is rarer with only conservative treatment to get good results.

Furthermore, the greater the damage, the less likely it is that the OCD heals on conservative treatment.

### 2.4 The localization of the osteochondritis

More than 70% of osteochondritis affects the internal femoral condyle posterior lateral part facing the intercondylar region. Osteochondritis located centrally on the lateral femoral condyle account for 15–20% of cases while damage to the trochlea is uncommon and occurs in less than 1%.^19^, ^20^, ^21^

Osteochondritis of the patella is also uncommon (5–10%) and is mostly located in the lower medial part. The medial changes are generally considerably smaller than those laterally located and thereby have a greater healing tendency.^19^, ^20^, ^21^

### 2.5 OCD treatment options

Because the natural course of those patients who have a stable osteochondritis is generally good if the physes are open an initial conservative non-operative treatment is most that is agreed upon.^22^^,^^23^

### 2.6 Non-operative treatment

A stable, juvenile OCD injury is usually treated by immobilization of the diseased joint with a locked brace or a brace that is laterally stable but allows some mobility. Useful orthoses for such treatment are the so-called Unloader orthoses which can be set in valgus or varus. One usually let the patient wear the orthosis for 6–12 months with partial loading and regular physical therapy.

If then the patient is pain-free at the 3-month check-up and if at the same time the x-rays show healing, you can let the patient load more, possibly start jogging. However, a more aggressive increase in activity should be stopped until several months of symptom freedom have passed. This is especially true activities that include jumping, torque, and heavy loading.

### 2.7 Operative treatment

One should consider operating on the patient when the osteochondritis is either.

1. Unstable, has loosened and in those patients with closed physes who did not respond to conservative treatments. The goal of an operation is to achieve congruity in the joint, a strong fixation of the fragment as well as repair of the entire osteochondral damage

Or.

2. Stable and injuries with partial detachment of fragments. This applies to OCD grade II and they can be treated arthroscopically with drilling the lesion to create channels into underlying bone that allows vascular ingrowth into the diseased bone to revitalize the bone and create healing conditions. The drilling can either take place via so-called antegrade or retrograde drilling direction.

#### 2.7.1 Accessibility

To evaluate if the OCD lesion is reachable via arthroscope, it is possible to use the simple ¨Cut doll¨-test. Print out a sagittal view from the MRI sequence of the OCD lesion and with a scissor cut out the side view of the femoral condyle. Use the cut out paper piece and rotate it against the remaining tibial plateau part to see the condyles accessibility (See Fig. 2A, Fig. 2BA–B).

**Fig. 2A:**
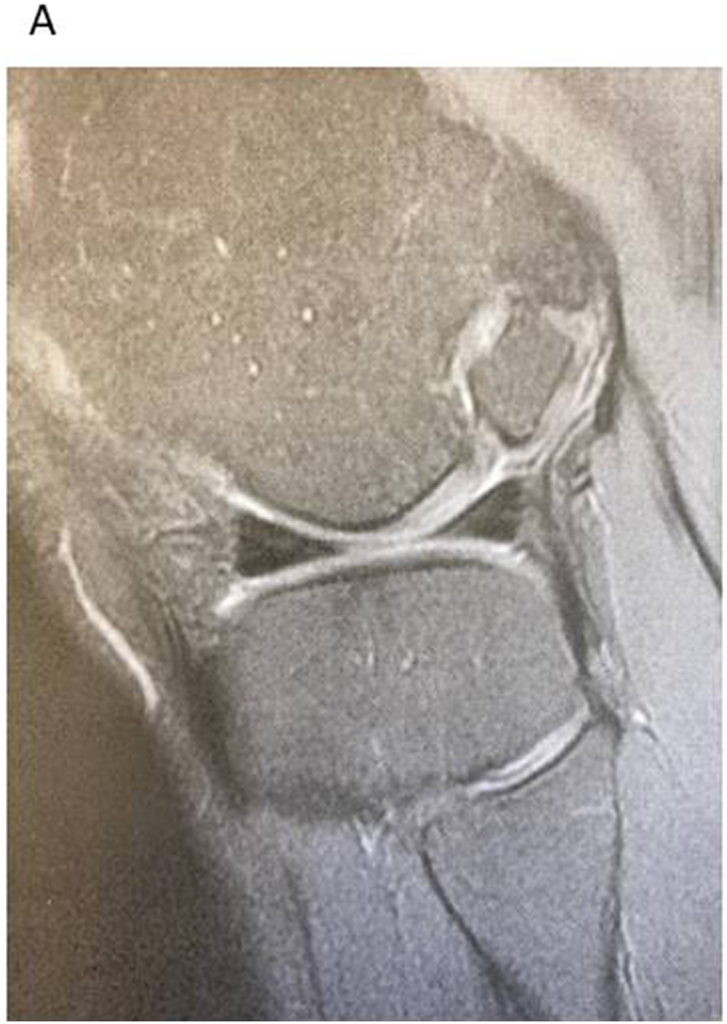
A large osteochondral fragment of an OCD situated quite posteriorly on the medial femoral condyle. The question is if it is reachable by *trans*-arthroscopic approach.

**Fig. 2B:**
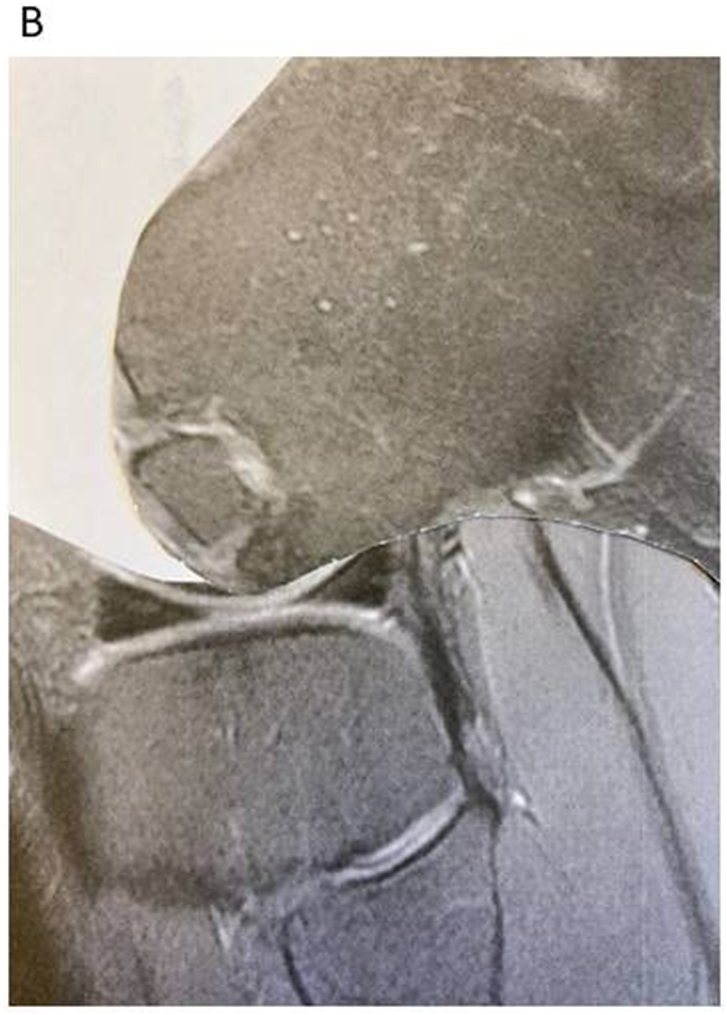
By printing out the sagittal MRI image and cut the image along the femoral condyle lining, one may rotate the cut area to make the image like a maximal flexion. By such a simple procedure, it is possible to get an idea if it is possible to use a trans arthroscopic intervention; the ¨Cut doll-test¨.

#### 2.7.2 Debridement

One option is to only remove loose parts of the OCD-lesion and clean the empty space. Such a careful debridement with removal of loose tissue may show good clinical results in the long term.^24^ However, Murray et al.^24^ found that after only fragment debridement, radiographic evidence of early degenerative joint disease in 17/24 (71%) of patients was found.^24^

#### 2.7.3 Antegrade epiphysis drilling

The antegrade drilling^25^^,^^26^ is done by drilling through the epiphysis to spare the joint surface. The technique is technically demanding but is made easier if you use a vector guide to get right into the spot. You use simultaneous X-ray translucency.

• If the lesions are located posteriorly on the lateral femoral condyle lesions, they are best seen with lateral fluoroscopy.
• Medial lesions are best seen with an anteroposterior view.

One uses k-threads and directs the first thread towards center of the lesion until it reaches into the bone but does not reach into the cartilage layer. It is also possible to use a special offset guide to spread the drill holes in the damaged area in a good way.

#### 2.7.4 Retrograd drilling

Here, you drill from inside the joint right through the cartilage layer to reach into the underlying bone.^25^^,^^26^ It is a technically easier method. Also with this methods, an offset can guide be useful for spreading the holes just right around the damaged area and 3–10 holes are reasonably to make related to the size of the damage. Bleeding through the boreholes indicates that one has passed the area of necrosis and reached into a well-vascularized bone marrow.

#### 2.7.5 Fragment fixation

Fragment fixation is used when there are ICRS Grade III-IV-lesions. The fixation of fragments is done via a mini-arthrotomy or trans arthroscopically. Unstable injuries must be carefully examined with arthroscopy for assessment whether it is realistic to try to heal the injury with fixation or whether the osteochondritis dissecans fragment should instead be removed. Most often the border zone between the fragment and surrounding normal cartilage can be seen as the fragment is elevated a bit making the border line visible. With the tip of the probe produce a slight pressure direct on that line and the tip of the probe will enter the border depth and the fragment may be slightly or fully uplifted. Through the opening, debridement of the bottom area may be done and drilling to vascularize the sclerotic bone area.

Small fragments from a larger piece can need to be removed while instead the larger fragment can be fixed.

Methods for fixating the fragments are as follows:

• Staples, Plain or threaded Steinman pins^27^^,^^28^
• Herbert screws or level countersunk cannulated screws^29^^,^^30^
• Absorbable pins^31^, ^32^, ^33^, ^34^
• Interpositional autogenous osteochondral grafts^35^
• Fibrin glue + sutures.^36^

The fixation can be done through.

• Fixating the osteochondritis when it is in place without moving it further.
• When the osteochondritis is loosened, the bone base is debrided and drilled before the osteochondritis is brought into place to be fixed.
• When a large osteochondritis is loosened with major bony involvement and depth more than 8 mm, the bone base is debrided, drilled plus the area is filled with bone graft from the proximal tibia or from the pelvis. Synthetic bone can also be mixed in here.

Important to notice is that K-wires do not cause compression and must be removed after the osteochondritis has healed. A Herbert screw allows a rigid fixation but also needs to be removed after bone healing. The resorbable pins do not need to be removed but they may cause reaction in the underlying bone with edema.

One can also use autologous osteochondral grafts to fix one osteochondritis with several sub fragments.^35^

However, often the osseous part of the osteochondritis is affected and has atrophied with dead bone, which means that it will fit poorly into the defect. If the bone area is of decent quality then you can try to fill the base of the bone with bone grafts and finally fit the osteochondritis fragment into the defect before fixation.

IIf the osseous component is of poor quality and thin, the healing prognosis is poor. It is then better to remove the osteochondritis fragment. The remaining crater is then cleared to stable vertical edges and the bone base is treated with bone marrow stimulation, preferably drilling to reach through the subchondral sclerosis and create revascularization and fibrous cartilage healing. Microfracture is not deep enough to reach the larger vessels needed to be connected to in order to get secure vascularization.

When the osteochondritis already is classified as ICRS Grade IV a reposition of loose fragment and refixation is less common. The fragment has often been loose for a long time and become deformed as well as having an insufficient underlying bone that makes re-fixation impossible.

In addition to the above mentioned bone marrow stimulation via drilling, there are other cartilage reparative methods to use as:

• Autologous cartilage cell implantation-grade 3 and 4. Generation 3 with in vitro expanded chondrocytes seeded in matrices are mainly useful for very large defects >3 cm^2^. ^37^ Smaller to medium sized lesions are possible to treat with autologous or allogeneic cartilage fragments such as Autograft^38^^,^^39^, CAIS^40^, CAFRIMA^41^ and PJAC^42^(See Fig. 3A, Fig. 3B, Fig. 3CA–C).

**Fig. 3A:**
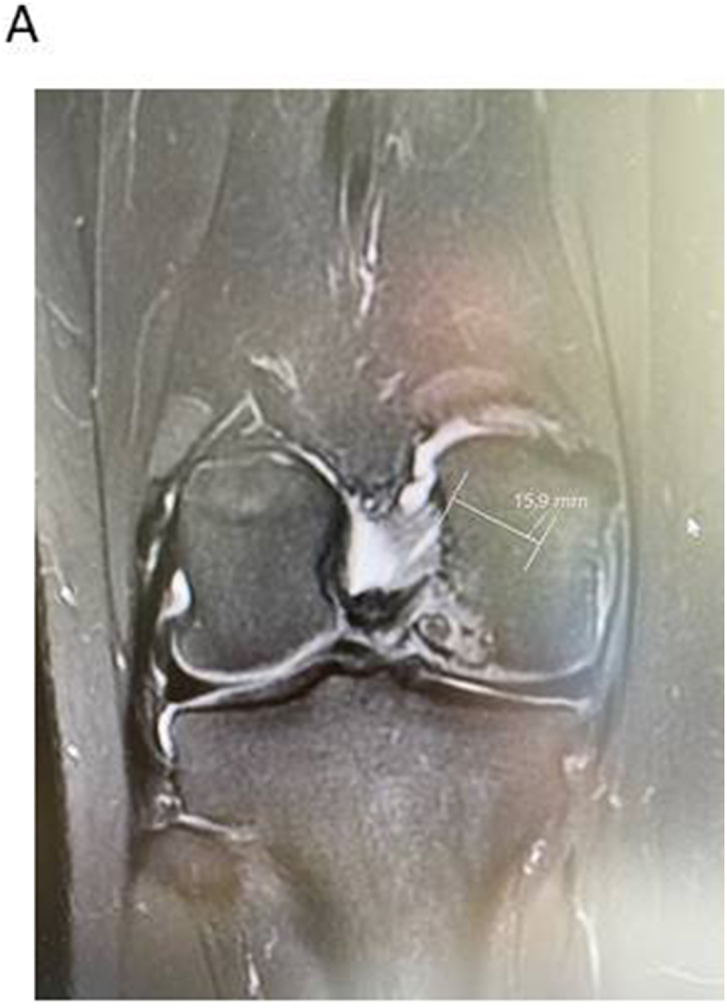
A coronal view of a medial femoral large OCD lesion.

**Fig. 3B:**
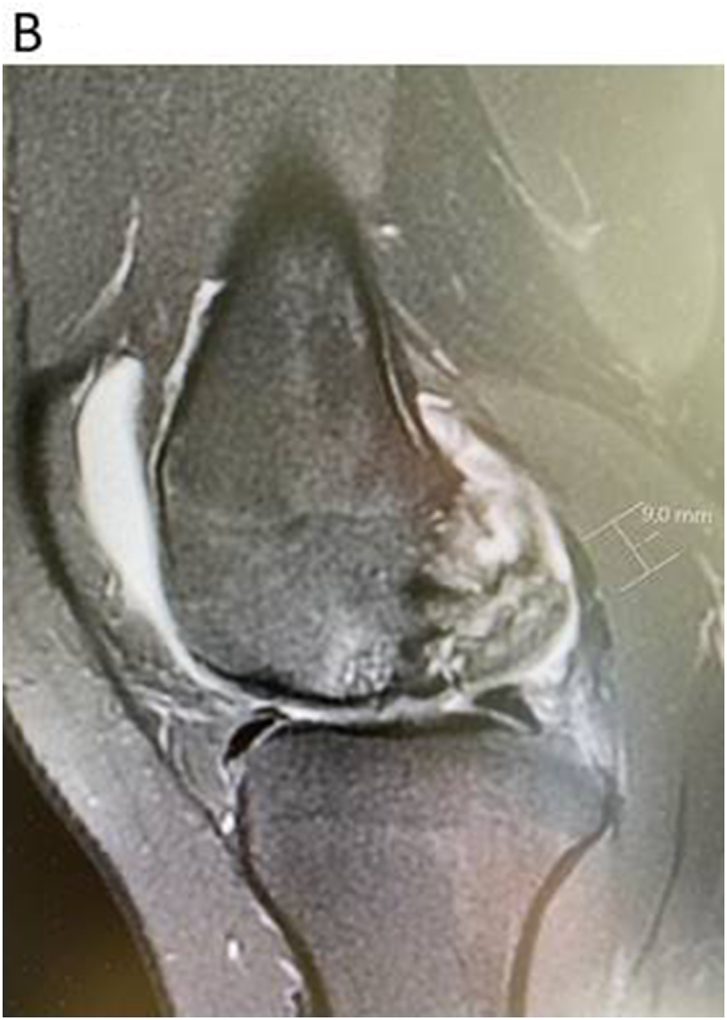
A sagittal view of the large femoral condyle OCD lesion.

**Fig. 3C:**
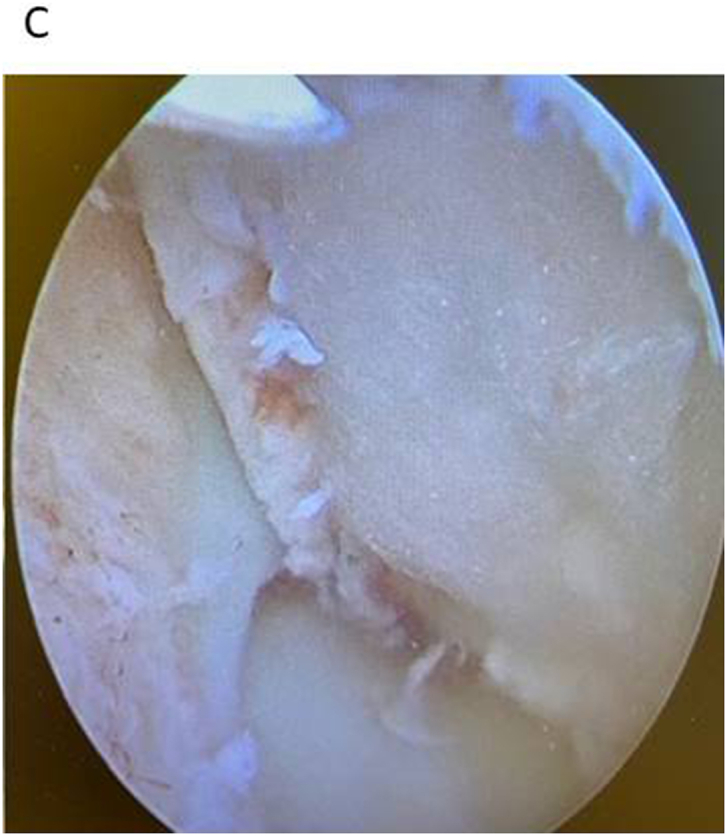
The large OCD lesion has been operated on. *Trans*-arthroscopic extirpation of the large osteochondral fragment with dead bone part + cartilage fragment implantation of the defect after debridement and drilling. The cartilage fragment have been harvested from the osteochondral loose piece, implanted in to the defect in fibrin glue and covered finally with a hyalyronic acid membrane.
• Mosaic plastic with autologous or synthetic plugs^43^^,^^44^
• Osteochondral Allografts^45^
• ¨Tissue engineering¨ with matrices and bone marrow materials such as carbon fiber, hyaluronic acid matrices and collagen matrices.^46^

If the bone depth is greater than 8 mm, it is recommended to use these methods combined with a simultaneous bone transplant.^47^ Most often bone grafts may be harvested from the proximal tibia.

## 3 A summary of what can be done

• Fixate the osteochondritis if the bony component is good and the overlying cartilage is intact. Which fixation is chosen depends on the skills and experience of the surgeon.
• If the osseous part of the osteochondritis is thin and sclerotic, then the healing prognosis is poor. Excise the osteochondritis fragment and perform a bone marrow stimulation and debridement in young patients. In slightly elderly, use more advanced cartilage repair techniques and if a deep defect is developed, combine with bone grafting.

## 4 Post-operative management

Give the patient a brace, locked in extension position for 2 weeks. Let the patient take load in the brace. Then on the 3rd week open the brace for a full range of motion and allow the patient to begin range of motion training successively for another 4 weeks.

If the damage is larger than 2 cm^2^, then let the patient continue with an unloader brace.

In the case of osteochondritis dissecans in an adult patient, more often an aggressive strategy is needed. In the still young patients <30 years of age still a bone marrow stimulation of an OCD IV may work.

Elderly patients with large defects in cartilage and bone after defect-healed OCD often requires additional unloading osteotomies.

## 5 Future research areas for improvement of OCD treatment

It is still unknown why an OCD develops even though causes that have been discussed are inflammatory and vascular origins plus repeated trauma.^48^ There seems also to be a hereditary/genetic possibility and it is important to notice similarities to other types of osteochondrosis like Legg-calve Perthes disease which develops at the hip joint, Panner's disease affecting the elbow, Freiberg's disease affecting the second toe and Köhler disease found on the foot. There seems to exist common triggering factors including stress to the bone, disturbances of blood supply to the affected area and trauma. A condition with similarities to human OCD is the type of osteochondrosis found in pigs and horse and it is the most frequent cause of leg weakness in rapidly growing pigs and leg lameness in horses. Osteochondrosis results in a local thickening of cartilage within the affected cartilage, which fails to ossify, retained in the subchondral bone. The retained cartilage is often associated with chondrocyte death and loss of extracellular matrix proteoglycans.^49^ An advanced degree of the condition is observed as osteochondrosis dissecans or secondary OA finally causing lameness of animals.^49^

In the animal studies, the identified genes tended to cluster at the protein secretion pathway influencing extracellular matrix molecules and finally the growth plate maturation.^50^ Similar to the animal osteochondroses is the familial cases of OCD that have been reported and also in some types of skeletal dysplasia. An early development of OA in those patients with familiar OCD has been associated by mutations in ACAN (19).^51^

We will see more of genome-wide association studies in the future in the identification of genes that increase the risk for osteochondritis dissecans (OCD).^49^ One part of the development of osteochondroses like OCD is a negative influence on vascularity. Growth cartilage has a temporary blood supply organised as end arteries. Vascular failure, infections and trauma can all obstruct those vessels. Early lesions of osteochondrosis are consistently found in regions where the temporary cartilage canal vessels traverse the chondro-osseous junction and a canal necrosis may start an osteochondroses.^52^Olstad et al.^53^ have shown that in foals, a subclinical stage of ischemic chondronecrosis exists that precedes and predisposes to the development of osteochondrosis dissecans and subchondral bone cysts.^53^

Future challenges are to differentiate between causes of vascular failure and to discover the nature of the heritable predisposition for osteochondrosis.^54^

Of interest is also the finding that a relative high percentage of patients with juvenile OCD also hade vitamin-deficiencies.^55^^,^^56^ Oberti et al.^55^ found that almost half of the patients diagnosed with JOCD presented abnormal serum levels of vitamin D. A two-fold incidence of vitamin D deficiency was found in patients that needed surgical treatment compared to patients treated conservatively.^55^Bruns et al.^56^ found that a vitamin D3 deficiency rather than an insufficiency may be involved in the development of OCD lesions. The authors stated that with a vitamin D3 substitution, the development of an advanced OCD stage may be avoided.^56^ Such a statement was supported by Fraissler et al.^57^ who found that vitamin D deficiency was frequent in patients with traumatic and idiopathic talus OCD. They believed it could be important to routinely assess and treat the vitamin D status of those patients.^57^

Three areas to consider for further research are subsequently:

The Genetic pathway.

The vascular pathway.

The nutritional pathway.

## Summary

Surgery should be considered in young patients with partially loose and unstable OCD and among the patients with closed physis who did not respond on conservative treatment.

A good clinical result can be expected when:

• The fuses are still open
• When the osteochondritis is small
• When the osteochondritis can be assessed as stable by MRI

But.

When you already see cracks and discontinuity in the cartilage layer or even a defect on the MRI with loose osteochondral fragments there, the prognosis is poor.

## Funding/sponsorship

To write this paper, the author did not receive any specific grant from funding agencies in the public, commercial or not-for-profit sectors.

## Informed consent

N/A.

## Institutional ethical committee approval

N/A.

## Authors’ contribution

The author is the only contributor of the paper.

## Declaration of competing interest

I have following potential conflicts of interest to disclose.

I am a member of the advisory board for Episurf Medical AB.

I have been on the speaker's bureau for Arthrex during 2021.

I am on the speaker's bureau for Anika

I have consultancies for Xintela AB and for Finceramica during 2021.

I am editor in chief of Journal CARTILAGE.
